# The RNA fold interactome of evolutionary conserved RNA structures in *S. cerevisiae*

**DOI:** 10.1038/s41467-020-16555-4

**Published:** 2020-06-03

**Authors:** Nuria Casas-Vila, Sergi Sayols, Lara Pérez-Martínez, Marion Scheibe, Falk Butter

**Affiliations:** Institute of Molecular Biology, Ackermannweg 4, 55128 Mainz, Germany

**Keywords:** Proteomics, Data integration, RNA metabolism

## Abstract

RNA-binding proteins play key roles in regulation of gene expression via recognition of structural features in RNA molecules. Here we apply a quantitative RNA pull-down approach to 186 evolutionary conserved RNA structures and report 162 interacting proteins. Unlike global RNA interactome capture, we associate individual RNA structures within messenger RNA with their interacting proteins. Of our binders 69% are known RNA-binding proteins, whereas some are previously unrelated to RNA binding and do not harbor canonical RNA-binding domains. While current knowledge about RNA-binding proteins relates to their functions at 5′ or 3′-UTRs, we report a significant number of them binding to RNA folds in the coding regions of mRNAs. Using an in vivo reporter screen and pulsed SILAC, we characterize a subset of mRNA-RBP pairs and thus connect structural RNA features to functionality. Ultimately, we here present a generic, scalable approach to interrogate the increasing number of RNA structural motifs.

## Abstract (web summary)

Previous study identified in vivo structured mRNA regions in *Saccharomyces cerevisiae* by dimethyl sulfate-sequencing. Here the authors use quantitative proteomics to identify protein interactors of 186 RNA folds in *S. cerevisiae*, providing functional links between RNA binding proteins and distinct mRNA fold.

## Introduction

RNA-binding proteins (RBPs) are key players in several aspects of co- and post-transcriptional gene expression regulation, namely RNA splicing, capping, polyadenylation, export, translation and turnover^1^. Although protein-centric methods are widely employed to study RNAs associated with preselected RBPs, RNA-centric methods coupled to mass spectrometry such as RNA pull-downs and RNA interactome capture (RIC) allow identification of protein interactors at a target RNA^2^. Previous polyA RIC studies have cataloged a comprehensive repertoire of RBPs in budding yeast, extending the known set of RBPs from previous low-throughput studies and in silico predictions based on similar RNA-binding domains (RBDs)^3–5^.

RNA can adopt complex structures critical for binding to proteins that can vary at different cellular environments. Thus, intensive efforts have been undertaken to investigate RNA folding. In this context, the in vivo identification of RNA structures has been facilitated by techniques such as selective 2′-hydroxyl acylation analyzed by primer extension (SHAPE) and dimethyl sulfate-sequencing (DMS-Seq)^6^. The latter was also employed to identify structured mRNA regions in budding yeast^7^. Subsequent phylogenetic analysis revealed that 188 of these in vivo structured mRNA regions were conserved among yeast species^7^. However, to further characterize these conserved putative protein-interacting structures a streamlined approach is required that can investigate dozens of short RNA fragments. Employing SILAC-based quantitative mass spectrometry, we map RBPs for this previous published set of evolutionary conserved RNA structures in yeast, extending the structural information to a functional context and thus providing a generic workflow to systematically study RNA–protein interactions at a large number of RNA folds harboring putative protein recognition elements.

Comparison with previous RBP datasets shows that a set of our RNA fold-associated proteins comprises well-studied RBPs for which we are able to reveal target mRNAs. Furthermore, we also report proteins with unassigned roles in RNA biology. Integration of genetic interaction data and experimental approaches to evaluate translational control provide first hints into the functional consequences of the reported mRNA–RBP interactions.

## Results

### Identification of proteins enriched at conserved RNA folds

**Fig. 1:**
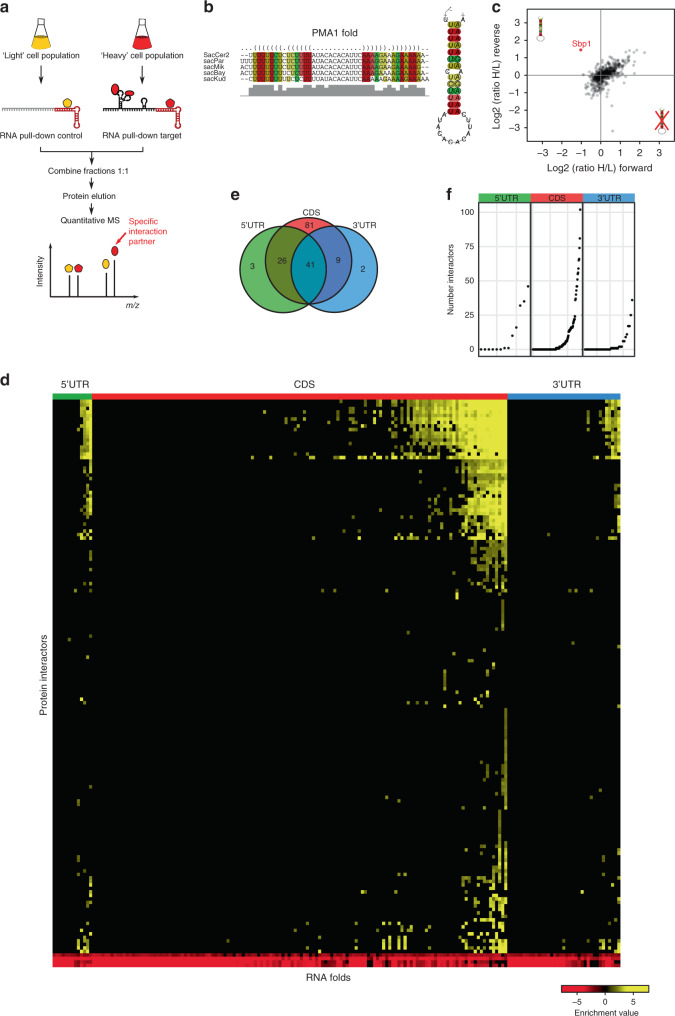
Workflow and results for identification of proteins enriched at evolutionary conserved RNA folds. **a** Schematic of the SILAC-based quantitative RNA–protein interactomics workflow. **b** Conservation analysis of the conserved fold in the *PMA1* mRNA by RNAz. Dots indicate unpaired bases and brackets paired bases. Gray bars represent sequence conservation among the indicated 5 yeast species. Folded structure shown on the right, with positional entropy values ranging from red/yellow (lower entropy) to green/blue (higher entropy). **c** Two-dimensional interaction plot comparing the interactors for *PMA1* wild-type hairpin with the mutated fold. **d** Heatmap showing enrichment values for the 162 protein-binding partners to the 186 investigated RNA folds. **e** Venn diagram showing overlap of interactors according to genomic position of the RNA fold (5′UTR, CDS, 3′UTR). **f** Dot-plot displaying the number of binders identified to each investigated RNA fold grouped by localization within the mRNA (13 5′UTR, 136 CDS and 37 3′UTR RNA folds) (Supplementary Data 2).

A recent study investigated mRNA structural features using DMS-Seq and revealed hundreds of in vivo and in vitro structured mRNA regions in *S. cerevisiae*^7^. In order to select for candidate structures with possible biological implications, a cut-off criteria was applied to DMS-Seq signal based on previously known functional structures like HAC1, RPS28B and ASH1. In addition, phylogenetic analysis on these mRNA structures revealed a list of 188 structured regions under positive evolutionary selection, lending additional support for a physiological function. These 188 evolutionary conserved RNA regions are of similar length, but found in different regions of the transcript (Supplementary Fig. 1a, b). We reasoned that one possibility for their strong evolutionary conservation is the recognition by RNA-binding proteins (RBPs). When we performed comparative analysis of the provided DMS-Seq datasets, we observed similar in vivo and in vitro folding features for this set of evolutionary conserved folds, suggesting a very robust intrinsic structural conformation (Supplementary Fig. 1c). This high in vitro/in vivo correlation indicated that our previously developed streamlined RNA–protein interaction screen based on quantitative proteomics^8^ is suited to identify putative protein binders to these RNA folds. We were able to transcribe 186 of the 188 conserved RNA folds with a S1-aptamer^9^ fused at their 3′-end for immobilization on a streptavidin matrix (Fig. 1a). Each of these RNA folds was compared to a generic control bait, a 161-bp 3′UTR *COX17* mRNA fragment harboring two Puf3-binding sites, in a quantitative SILAC-based RNA pull-down. To this end, the fold and the control were incubated with differentially labeled SILAC-encoded extract from *S. cerevisiae*. In order to reduce the number of competing unspecific binders, a pool of all investigated RNA folds without the S1-aptamer was added to each pull-down (Supplementary Fig. 1d).

In a proof of concept, we applied this workflow to a functionally validated hairpin structure in the 5′UTR of the *PMA1* mRNA^7^. This structure is under positive evolutionary selection as exemplified by compensatory mutations in other yeast species (Fig. 1b). Disruption of the stem loop by non-compensatory mutations is known to change gene expression^7^. To explore a possible regulation by RNA-binding proteins, we determined binding partners to this structure and applied our quantitative proteomics workflow comparing the wild-type *PMA1* hairpin to a mutated dysfunctional structure. In this experiment, we identified Sbp1, a known translation repressor, enriched at the wild-type *PMA1* structure, attesting our ability to identify protein-binding partners to RNA structures (Fig. 1c). We conducted the screen (744 pull-downs) in a label-switch fashion, resulting in a forward and reverse experiment for each query RNA fold^8^. We compared two strategies to filter for enriched proteins, one with a flexible cut-off depending on the enrichment of the known binder Puf3 on the control bait and the other based on a log2 SILAC ratio > 1, representing a two-fold enrichment (Supplementary Fig. 1e). In order to ensure technical quality and reproducibility in our streamlined screen, we monitored the enrichment of the known interactor Puf3 at the *COX17* RNA fragment together with three other repeatedly binding proteins (Lsg1, Sui3 and Gcd11) (Supplementary Fig. 1f). Requiring a stringent filter of at least two-fold enrichment against the control RNA in both forward and reverse experiments, we identified 162 proteins interacting with the investigated 186 conserved RNA folds (Fig. 1d and Supplementary Data 1). Notably, the length of the RNA fragment did not correlate with the number of bound proteins, excluding a systematic bias as would be apparent for unspecific background (Supplementary Fig. 1g). In fact, the number of interacting proteins per mRNA fold fragment is quite diverse (Supplementary Fig. 1h). Although 25% of our interactors (*n* = 41) were enriched at folds irrespective of the functional region of the mRNA (5′UTR, CDS, 3′UTR), 50% of them showed positional binding preferences (*n* = 82) (Fig. 1e). Notably, none of our interactors showed exclusive simultaneous binding to the 5′ and the 3′UTR, indicating a strong functional separation of the two different UTRs (Fig. 1e). Irrespective of their genomic location, for 42% of the RNA folds we did not detect an interaction partner; however, we observe a preference for CDS RNA folds to present a higher number of interactors (Fig. 1f). Some RNA folds in our dataset are partially overlapping in sequence within the same mRNA. A comparative analysis on the interactors of partially overlapping RNA folds can help delimit the relevant sequence for a given protein–RNA interaction. Our interactomics data cover 36 mRNAs with multiple RNA folds (2–6 folds per mRNA) and thus can be used to gain information on the localization specificity of our interactors. A specific analysis on *SSC1* and *YNL190W* mRNAs shows that RNA folds at different positions along the mRNA have a different set of interacting proteins, allowing us to describe the binding position of these interactors (Supplementary Fig. 1i).

### Correlating our interactor set with RBP features

**Fig. 2:**
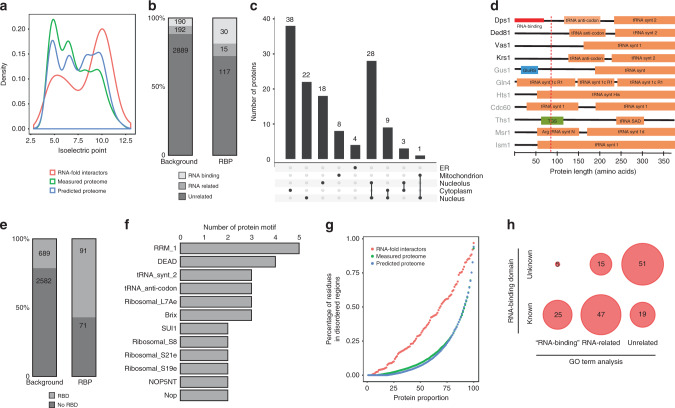
Enrichment of RBP features in our RNA fold interactome. **a** Isoelectric point density of the RNA fold interactome (orange), measured proteome (green) and the predicted proteome (blue) shows bias for RBPs. **b** Classification of RBP candidates based on GO-term analysis. Comparison of the measured proteome (background) and the RNA fold interactome. Enrichment of RNA binding and RNA-related GO terms was calculated with Fisher test odds ratio = 2.9, *p*-value = 6.2e^−08^. **c** Classification of the interactors based on their cellular localization. **d** Schematic of tRNA synthetases domain organization. tRNA synthetases identified in our screen are annotated in black. **e** Count of RBP candidates with RNA-related annotated Pfam protein domain (Fisher test odds ratio = 4.80, *p*-value < 2.2e^−16^). Shown are the measured proteome (background) and the RNA fold interactome. **f** 12 most abundant RBDs of our RNA fold interactome. **g** Distribution of predicted disordered regions for the RNA fold interactome (orange), measured proteome (green) and predicted proteome (blue) shows enrichment among RBPs. **h** Characterization of RNA fold interactome by GO annotations and protein domain-based classification.

We first inspected the biochemical properties of our RBP candidate set to exclude putative technical bias in MS measurement. Neither for the measured proteome nor for the RNA fold interactors did we observe a substantial bias for protein size, length and hydrophobicity when compared to all yeast proteins (predicted proteome) (Supplementary Fig. 2a). However, for the RNA fold interactors, we noted a significant shift on the isoelectric point distribution, implying that basic proteins are more prevalent in our interactor set compared to the measured proteome, a distinctive feature of known RBPs (Fig. 2a)^10^. Consistent with this observation, we found a high enrichment of the basic amino acids lysine, and to some extend also for arginine, in the amino acid composition of our RNA-binding proteins (Supplementary Fig. 2a). 25% of our associated RBPs seem to be very specific and recognize a single RNA fold, whereas a significant fraction (24 out of 186) show a promiscuous binding ability, ranging from 20 to 90 target folds (Supplementary Fig. 2e). For instance, Yra1 is among the selective RBPs, detected highly enriched at the two RNA folds on *FAS2* and *SSE1* mRNAs. As it is known that Yra1 drives mRNA export and requires the Dbp2 helicase to unwind RNA^11^, our data show co-enrichment of Dbp9, another DEAD-box RNA helicase that has previously been implicated in rRNA processing^12^. Our co-enrichment, together with the recent identification of Dbp9 as an mRNA-binding protein by other studies^3^, might point to a potential coordinated function of Yra1 with Dbp9, in addition to the previously known function with Dbp2.

There is no correlation between the protein abundance and the binding of the protein to our RNA folds (Supplementary Fig. 2b). We also explored whether high abundant proteins would be biased to bind multiple folds or specific fold types (5′UTR, CDS, 3′UTR) and observed no correlation (Supplementary Fig. 2c). GO-term enrichment analysis on our interactors ranked by genomic position of the fold (5′UTR, CDS or 3′UTR) revealed enrichment of GTPase activity involved in translation initiation for the 5′UTR interactors, structural constituents of the ribosome and helicase activity for CDS-binding interactors and prevalence of nuclease activity involved in mRNA catabolism for 3′UTR interacting proteins (Supplementary Fig. 2d and Supplementary Data 6).

To further characterize our RNA fold interactor dataset, we integrated data from public repositories and previous global RIC screens. As expected, our interactors are enriched for the gene ontology term RNA binding (Fig. 2b). As our approach also identifies proteins that are not in direct contact with RNA, e.g. as part of an RNA-binding protein complex, by using GO annotation, we classify 30 of our 162 interactors as previously reported RNA-binding proteins, such as Bfr1, involved in localization of mRNAs to P bodies^13^. When we classify our interactors according to their cellular localization, we find that 24% are exclusively found in the cytoplasm and approximately 50% relate to nuclear functions (Fig. 2c), whereas a few of them localize to mitochondria.

To allow for a more comprehensive examination of our RBP candidates, we extended our computational analysis to include proteins with related GO terms such as tRNA- and rRNA-binding (Supplementary Data 2). This increased the number of proteins with a known RNA-related functionality to 45, representing a nearly 3-fold enrichment compared to the reference proteome (Fig. 2b).

This includes Dbp3, a RNA-dependent ATPase involved in rRNA cleavage^14^ that has previously been reported as an mRNA-binding protein by other RIC studies^3–5^, four yeast tRNA synthetases Dps1, Ded81, Vas1 and Krs1 and the tRNA ligase Trl1. Examples of tRNA synthetases with a transcript-selective translation control function have been described before in vertebrates and yeast^15,16^. The N-terminus of Dps1 harbors an RNA-binding motif that enables binding to the 5′-end of its own mRNA and thereby inhibits its own translation^15^ (Fig. 2d). We find that Dps1 binds to 24 of our 186 investigated RNA folds suggesting a broader translational regulation than just its own mRNA. Indeed, RNA folds bound by Dps1 are primarily located on mRNAs that represent genes with well-defined functions in amino acid synthesis pathways such as MET6, ILV2, ARG1, HIS3 and GUS1^17–21^. Perhaps, in cellular conditions of low amino acid concentrations and impeded tRNA loading, Dps1 becomes available to bind to the conserved RNA folds in the mRNA of these amino acid metabolism genes and thereby controls amino acid synthesis as already suggested for other enigmRBPs^4^. Of note, the three other tRNA synthetases in our interactome (Ded81, Vas1 and Krs1) bind to a small, but highly overlapping set of RNA folds (Supplementary Fig. 2f). Interestingly, the target mRNAs of these three proteins are functionally related, as they are involved in protein folding, protein targeting and endocytosis (represented by SSC1, SSE1, VMA3 and SUR7)^22–25^. This suggests that the possible secondary activity of tRNA synthetases as translation regulators might not be restricted to Dps1. Supporting this idea and in contrast to other yeast tRNA synthetases, the four tRNA synthetases identified in this screen are characterized by a disordered N-terminal extension that might function as an RNA-binding domain^26^ (Fig. 2d).

We also analyzed the occurrence of classical RNA-binding domains (RBD). To this end, we used Pfam annotations and also evaluated RNA-binding domains from a manual curated dataset^1^. We find that 91 of our interactors have a known RNA-binding domain (Fig. 2e). The three previous global RIC studies in *S. cerevisiae* together reported only 48 of these binders while 43 are unique to this study.

Besides the common RBD domains (RRM, DEAD, tRNA- and ribosomal-related) (Fig. 2f), we also identify proteins with non-canonical RNA-binding domains. For example, Tma20 binds 11 different RNA folds, has an unknown function but associates with ribosomes, contains a PUA domain and has not been reported by previous interactome capture studies^27^. Proteins with PUA domains might represent a novel type of translation factors, since this domain was detected in proteins that also harbor domains homologous to the translation initiation factors eIF1/SUI1^28,29^. Consistently, Tma20 is homologous to the human MCT-1 gene that functions as a translation initiation factor^30^. At 8 of our 11 RNA folds targeted by Tma20, we also enrich Tma22, a protein with unknown function, but similar to the human DRP1 protein and possibly linked to translation regulation via its SUI1 domain. Our observations, together with previous studies reporting a physical interaction for these two proteins^7^, suggest a possible joint function for Tma20/Tma22 at their target mRNAs. Further experiments are needed to unravel a possible coordinated Tma20/Tma22 function on translation regulation.

Although RBPs have historically been associated with structured RBDs, recent studies revealed protein binding to RNA through intrinsically disordered and low amino acid complexity regions^26^. In this line, a previous interactome capture study from mESCs reported enrichment of low-complexity and disordered regions on their RBP candidates and suggested it to be a general feature of RBPs^31^. Indeed, we also report significant enrichment of disordered region containing proteins on our interactor dataset, further substantiating this as a possible feature of RNA-binding proteins (Fig. 2g).

51 of our 162 identified interactors still remain unexplained in the context of RNA associated proteins in *S. cerevisiae* (Fig. 2h). Six of these have human orthologues related to RNA biology by GO terms analysis, suggesting a possible RNA function also in yeast. Others, like inosine monophosphate dehydrogenase enzymes (IMD1, IMD2, IMD3) have also been reported in global RIC studies of *S. cerevisiae*^32^ and thus may belong to the class of enigmRBPs. Although we are not able to clearly identify direct RNA-binding proteins in our setup, most of the additional candidates might also be proteins associating to mRNA in form of complex members, extending the set of proteins involved in RNA regulation beyond the direct interactors reported by RIC (Supplementary Fig. 2g).

### In vivo validation of mRNA–RBP interactions

**Fig. 3:**
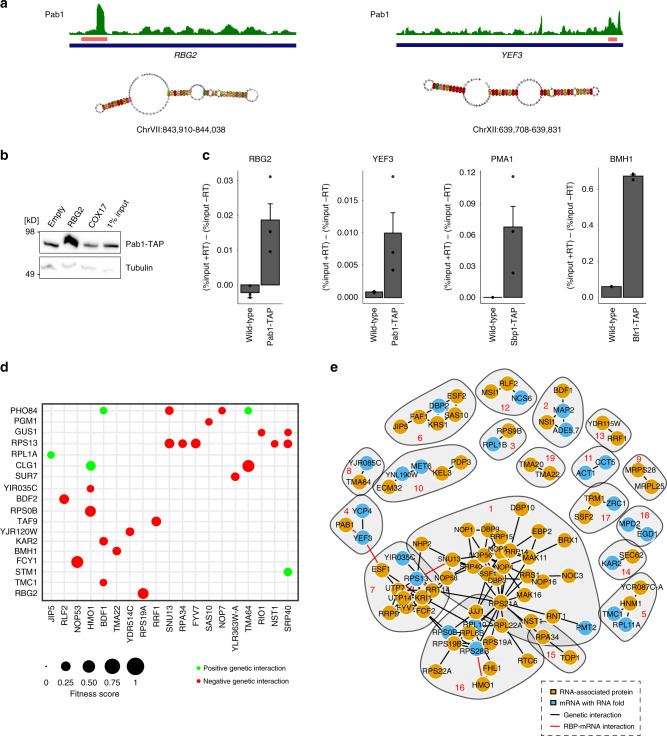
Integration of in vivo localization and genome-wide genetic interaction data provide functional insights into our set of interactors. **a** Pab1 PAR-CLIP data show a significant peak on both *RBG2* (*p*-value = 1.5e^−14^) and *YEF3* (*p*-value = 9.1e^−9^) target genes and overlap with the conserved RNA fold region (colored salmon). Folded structures are shown, with positional entropy values ranging from red/yellow (lower entropy) to green/blue (higher entropy). **b** Pull-down and Western blotting on a TAP-tagged Pab1 strain validated Pab1 binding to the *RBG2* RNA fold. **c** RNA immunoprecipitation (RIP) experiments in wild-type and endogenously TAP-tagged strains show enrichment of target RNAs in Pab1 (for *RBG2* and *YEF3*), Sbp1 (for *PMA1*) and Bfr1 (for *BMH1*). Results are shown relative to input signals normalized to the −RT (no reverse transcriptase) conditions. Data are presented as mean ±SEM values in *n* = 3 technical replicates. **d** Matrix showing genetic interactions described for our RBP (*x* axis) and RNA fold (*y* axis) interacting pairs. Genetic interactions with a fitness score > 0.08 are colored according to positive (green) and negative (red) interactions. The circle size is proportional to the fitness score of the double knock-out strain of the two relevant genes. **e** Clustering of our RBP candidate genes and mRNA genes according to the genetic interaction profile correlations (similar genetic interaction profiles considered upon PCC values > 0.2). Red numbers indicate the ID of the respective gene community (Supplementary Data 4).

We made use of the few available PAR-CLIP data in *S. cerevisiae* to validate our RBP-RNA fold interactions^33^. PAR-CLIP data for Pab1 is consistent with our results showing Pab1 recognition of RNA folds at the *YEF3* and the *RBG2* mRNA (Fig. 3a). We validated this interaction using a TAP-tagged Pab1 strain for the RNA pull-down (Fig. 3b). Interestingly, in both cases Pab1 binds to RNA folds in the coding region of its target mRNAs. We used two additional PAR-CLIP datasets for Nab2 and Yra1 to validate even less strong enrichment found for RNA folds within the *RBG2* and *TMC1* mRNA (Supplementary Fig. 3a). Extending our validation, we performed RNA immunoprecipitation to validate a few more protein–RNA interactions using available TAP-tagged proteins: Pab1 to *YEF3* and *RBG2*, Sbp1 to *PMA1* and Bfr1 to *BMH1* (Fig. 3c). Overall, these selected examples underscore nicely that we indeed identified in vivo relevant RBP–mRNA interactions.

### Functional hints from genetic interaction data integration

In order to explore functional relationships between our RBP candidates and their target mRNAs, we made use of recent whole genome yeast genetic interaction data^34^. Whereas the genetic interactions suggest synergistic effects of genes working in compensatory pathways, combination with our interactomics dataset can point to a possible mechanistic model. Our analysis resulted in 27 positive or negative genetic interactions of our RBP–mRNA pairs that allow speculation of mechanistic links (Fig. 3d and Supplementary Fig. 3b). For example, *TMA64* presents a positive genetic interaction with *PHO84* and additionally, we detected Tma64 enriched at the evolutionary conserved RNA fold on *PHO84* mRNA that encodes for an inorganic phosphate transporter (Supplementary Fig. 3c). Tma64 is not described at a functional level; however, it harbors a putative RNA-binding domain and has been linked to translation control^32,35^. In addition, *TMA64* presents another genetic interaction with the *CLG1* gene and we report it as interactor of an RNA fold on the *CLG1* mRNA. Clg1 is a cyclin-like protein that exerts its function through the interaction with Pho85, a cyclin-dependent kinase linked to phosphate response^36^. Specifically, under high phosphate conditions, the Pho85–Pho80 complex phosphorylates the transcription factor Pho4, promoting its nuclear export and thereby preventing transcription of genes related to phosphate starvation^37^ (Supplementary Fig. 3c). In addition, we report Tma64 enrichment at a 5′UTR RNA fold of the *PCL5* mRNA, encoding for yet another cyclin that is phosphorylated by Pho85 and involved in the amino acid starvation response^38^ (Supplementary Fig. 3c). Altogether, collective evidences from our interactomics screen and the genome-wide genetic interaction data insinuate a role for Tma64 in phosphate homeostasis, perhaps regulating the expression of starvation-related genes via recognition of structural features on its target mRNAs.

To further examine functional connections between our RBP and mRNA folds, we clustered them based on similarity in their large-scale genetic interaction profile and tested for enriched GO annotations in each group (Fig. 3e and Supplementary Fig. 3d). Genes that belong to the same pathway often share phenotypes with their target genes and therefore share similar genetic interaction profiles. For example, the central densely connected nodes mainly consisting of RBP candidates are characterized by ribosomal-related functions such as rRNA processing and ribosome biogenesis (communities 1, 7, 16). Also, other clusters present mitochondrial translation (community 9) or chromatin organization (community 2) functions. Similarly, the membership of uncharacterized RBP candidates to the resulting communities can be used to infer putative functions. For instance, in addition to the in vivo validated binding of Pab1 to the conserved RNA fold on the *YEF3* mRNA (Fig. 3a); a functional connection between *PAB1* and *YEF3* mRNA is further supported by similar genetic interaction profiles (community 4) (Fig. 3e). In another case, *YDR115W* clusters together with the mitochondrial ribosome-recycling factor *RRF1*, perhaps suggesting a possible role for YDR115W in mitochondrial translation (community 13). As shown above, our integration with physical interactomics data can provide hints towards a putative regulation mechanism of genes that share similar genetic interaction profiles.

### Translational control by RBPs binding to mRNA folds

**Fig. 4:**
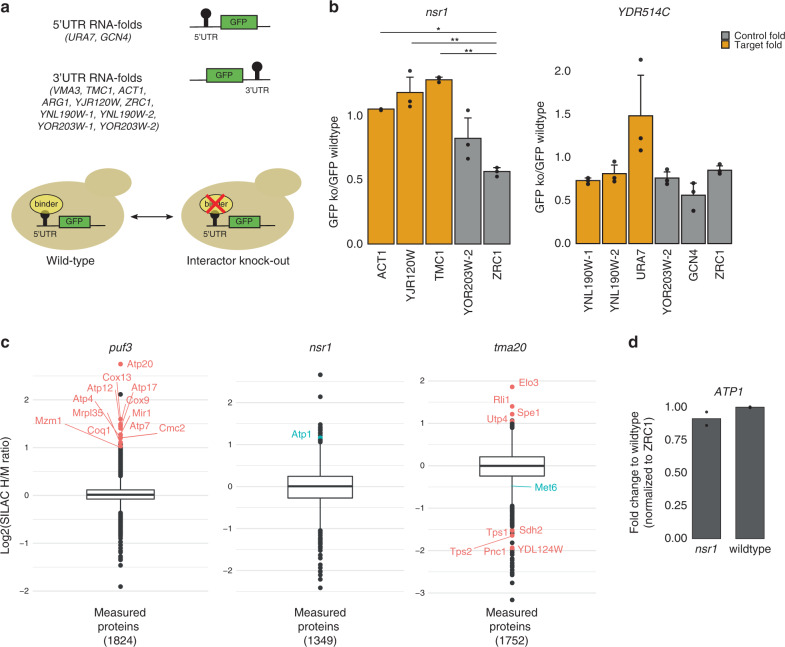
Functional validation of mRNA–RBP interactions. **a** Schematics of the GFP reporter screen with either a 5′UTR or 3′UTR fused RNA fold. Expression is compared between the wild-type and knock-out yeast strain of the respective interactor. **b** Bar-plots display changes on the reporter expression levels when Nsr1 and YDR514C are bound to their respective folds (orange). Control experiments are RNA folds with no detected Nsr1 or YDR514C binding (gray). *p*-value * < 0.05 and ** < 0.01 based on analysis of variance (two-sided ANOVA and Tukey post-hoc test) in *n* = 3 biologically independent samples. ACT *p*-value = 0.017, YJR120W *p*-value = 0.005 and TMC1 *p*-value = 0.003. Data are presented in mean ±SD values. **c** Pulsed SILAC box plots showing log2(SILAC H/M ratio) values of all measured proteins for *puf3* knock-out, *nsr1* knock-out and *tma20* knock-out strains compared to a wild-type strain (Supplementary Data 7). Boxes show median (center) and interquartile ranges (ends), lower whisker representing the smallest observation greater than or equal to 1.5 times the interquartile range and upper whisker representing the largest observation less than or equal to 1.5 times the interquartile range. **d** qRT-PCR expression levels of *ATP1* mRNA in a *nsr1* knock-out strain compared to the wild-type strain. Data are presented as mean values in *n* = 2 technical replicates.

To explore the biological consequences of our identified RBP-RNA fold pairs experimentally, we first focused on possible translational regulatory effects exerted by RNA folds in UTR regions. We incorporated two 5′UTR- and ten 3′UTR-folds at the respective untranslated regions of a GFP reporter construct and quantified changes in GFP expression by fluorescence intensities upon knock-out of the respective interacting RBPs (Fig. 4a and Supplementary Fig 4a). We validated our strategy with a known functional 5′UTR fold *SFT2*^7^. Consistent with previous data, disruption of base-pairing interactions in the 5′-*SFT2* hairpin resulted in an increase of GFP levels, demonstrating a repressive function of the *SFT2* hairpin on mRNA expression (Supplementary Fig. 4b). We applied this workflow to our 12 different UTR-folds, which were investigated in the relevant yeast knock-out strains and compared to wild-type (Fig. 4a). We quantified reporter levels of all 73 possible combinations of these 12 RNA folds and their identified interactors under normal growth conditions. Two RNA folds without any identified interactor (Fig. 1c) were used as controls. As transcriptional regulation is only one possible regulatory scenario that can be executed by our RNA fold interactors, we found two of them Nsr1 and YDR514C, that showed expression changes of the reporter construct (Fig. 4b). The yet uncharacterized protein YDR514C that bound to the RNA fold in the 5′UTR of the *URA7* mRNA behaves like a translational repressor in our screen, identical to Nsr1. As YDR514C has no annotated domains, Nsr1 harbors two RNA recognition motifs (RRM) and has been reported to be involved in rRNA processing^39^. In our screen, we detected Nsr1 binding to 60 of our RNA folds (Fig. 1d) and tested three of these RNA folds found in the *ACT1*, *YJR120W* and *TMC1* mRNAs, for a putative translational regulation. Concomitant with a role in gene expression, we observed an increase in reporter levels for the three Nsr1 target 3′UTR RNA folds upon knock-out of Nsr1, while at two control RNA folds that did not interact with Nsr1, we did not observe increased GFP expression.

We also explored a putative role of selected protein–RNA fold pairs on the translation of their target mRNAs by pulsed SILAC^40^. This technique determines protein synthesis and turnover rates via the differential incorporation of isotopically labeled amino acids. We thus compared global protein translation rates between wild-type and selected strains where our interactor was deleted. We used Puf3, a protein known to promote mRNA degradation of nuclear-encoded mitochondrial proteins^41^ as a positive control and indeed observed upregulation of the reported Puf3 target proteins in the Puf3 knock-out strains (Supplementary Fig. 4c). We additionally detected upregulation of 12 additional mitochondrial proteins (Fig. 4c). In 6 cases their mRNAs contain a single or multiple copies of the canonical Puf3-binding motif (UGUAAAUA)^42^ in their 5′UTR, CDS or 3′UTR (Table 1).

**Table 1:** Motif analysis on Puf3 targets.

mRNA	UGUAAAUA motif	Motif location	Similar motifs
COX13		3′UTR	UGUAAA
COX9			
MRPL35	UGUAAAUA	CDS	
ATP12	UGUAAAUA	3′UTR	
ATP7		3′UTR	GUAAAUA
ATP17	UGUAAAUA	5′UTR	
ATP20		3′UTR	GUAAAUA
ATP4			
MZM1	UGUAAAUA	3′UTR	
COQ1	UGUAAAUA	3′UTR	
MIR1		3′UTR	UGUAAAU
CMC2	UGUAAAUA	5′UTR/3′UTR	

In line with our GFP reporter screen data, pulsed SILAC experiments showed a translational repression role of Nsr1 on its target *ATP1* (Fig. 4c), as no changes were observed at the mRNA level (Fig. 4d). In agreement with our observations, MS studies^32,43^ captured Nsr1 as a physical interactor of proteins with a defined role in post-transcriptional gene regulation such as the RNA helicase Dbp2^11^, the translation initiation factor Ded1^44,45^ and the RNA poly(A) tail-binding protein Pab1^46^.

We also investigated Tma20 by pulsed SILAC, the protein we found in our screen to be binding to several RNA folds and that is homologous to the human MCT-1 translational regulator. We found proteins involved in different cellular metabolic processes related to carbohydrates (Tps1, Tps2), nucleotides (Pnc1), amides (YDL124W), acetyl-CoA (Sdh2) and amino acids (Met6) to be upregulated in its knock-out strain compared to wild-type. These data establish Tma20 is a positive translational regulator as suggested before based on our interactome data (Fig. 1d).

## Discussion

In this study, we have used a streamlined RNA interactomics strategy to map protein interactors to 186 evolutionary conserved RNA folds in *S. cerevisiae*. We showed that our interactor set was enriched for RBP features and fused our interactomics data with genome-wide genetic interaction data to suggest putative functional and mechanistic insights for the detected mRNA–RBP pairs. Finally, we explored translational regulation as a possible functionality of our mRNA–RBP interactions using a reporter screen system and pulsed SILAC.

As current approaches to study RNA–protein interactions such as RIC globally address RBPs binding to mRNA and polyadenylated lncRNA, we here map 162 RBP interactors to individual RNA folds. We show that our setup captures interactions at these RNA structures even for low abundant RBPs as judged by their low abundance in our measured proteome and that were missed by RIC experiments. Our approach does not only capture proteins in direct contact with RNAs but with the current washing conditions also enriches for RBP complexes. To quantify this ratio based on our analysis, 69% are known direct RBPs, whereas the remaining might be associated via protein-protein interactions.

We used available PAR-CLIP *S. cerevisiae* datasets to validate our detected RNA fold–RBP interactions. Although the number of PAR-CLIP datasets in yeast is extremely limited, the available studies show a very high overlap to our data attesting our ability to report physiologically relevant interactions. We furthermore validated experimentally more mRNA–RBP interactions by RNA immunoprecipitation ourselves.

Current knowledge about RBPs involved in post-transcriptional regulation focuses on either the 5′ or 3′UTR sequences. Interestingly, a significant portion of the conserved RNA folds is found in the CDS of mRNAs. We here report a significant number of RBPs recognizing RNA folds in the CDS of mRNAs. These interactors are related to ribosomal biosynthesis, tRNA binding or are metabolic enzymes and kinases. In addition, the binding profile of some classical RNA-binding proteins can be surprising, such as the binding of Pab1 to CDS RNA folds such as *YEF3* and *RBG2* (cross-validated by PAR-CLIP data). These observations suggest that RNA-binding motifs may also form in the coding region, possibly allowed by the degenerated codons. Whether this binding results in functional consequences will need to be explored in the future.

As noted in previous RIC experiments already, not all discovered RBPs harbor canonical RBDs, but also include known proteins with diverse cellular functions like kinases, metabolic enzymes and tRNA- and rRNA-metabolism factors^10^. Using the resolution for individual RNA folds, we here showed that tRNA synthetases bind to a specific set of RNA folds. This observation immediately suggests that some tRNA synthetases harbor yet unknown RNA-binding domains and hint to a possible role as translation regulators.

Overall, we here outlined how smaller structural features within an RNA molecule can be investigated with a streamlined assay, resulting in the identification of 162 interactors to 186 evaluated RNA folds. Mutational studies should be the next step to characterize our set of protein–RNA fold interactions to decipher the required RNA-binding motifs in more detail. There will be a growing demand for such analyses as thousands of structures can nowadays be either obtained using in vivo RNA structure methods, like DMS-Seq or SHAPE, functional reporter assays or computationally predicted based on conformation energy^47^.

## Methods

### DMS-Seq data comparison for conserved RNA folds

DMS-Seq datasets from three different experimental conditions (in vivo, in vitro and denatured) were retrieved from GSE45803^7^. DMS signals of the corresponding genomic loci for each RNA fold were retrieved and normalized proportionally to the most reactive base within a given structure. For each RNA fold, denatured DMS signal was subtracted to the in vivo and in vitro DMS signals and Spearman correlations between the resulting in vivo and in vitro DMS signals were calculated as a measure of similarity. The probably of each RNA fold to form structure was calculated based on the Gini coefficient. This coefficient defines inequality within a population, which in our case means that RNA folds with high probability for structure formation will have very unequal DMS signal distribution along the sequence (Gini coefficient ≅ 1), whereas folds with low structural formation capacity will have an even DMS signal distribution (Gini coefficient ≅ 0). The resulting Gini coefficients of the conserved RNA folds (*n* = 188) were compared to the Gini coefficients of 200 randomly picked regions of similar average length from different genomic locations (intergenic, 5′UTR, 3′UTR, CDS).

### RNA conservation analysis

*PMA1* RNA fold sequences for *S. cerevisiae*, *S. paradoxus*, *S. mikatae*, *S. kudriavzevii*, *S. bayanus* were obtained from UCSC (SacCer_Apr2011/SacCer3 assembly) and aligned with ClustalW version 1.81^48^. The resulting multiple sequence alignment was fed into RNAalifold 2.2.8 from the Vienna RNA package^49^ to predict the consensus secondary structure.

### Yeast genomic DNA extraction

Yeast genomic DNA from BY4741 strain was extracted from a 2 mL saturated culture, centrifuged (2000×*g*, 5 min) and resuspended in Lyticase buffer (1 M Sorbitol, 100 mM EDTA pH 8, 14.3 mM beta-mercaptoethanol). After 1 h incubation at 37 °C with 2.5 μL lyticase (Zymolyase® 20 T (≥20 U mg^−1^)), samples were centrifuged (5000 rpm, 5 min) and pellets washed twice with 1 mL spheroblast wash buffer (1 M Sorbitol, 100 mM EDTA pH 8). Pellets were then resuspended in 500 μL TE 50/100 (50 mM Tris-HCl pH 7.5, 100 mM EDTA pH 8) and incubated with 50 μL 10% SDS for 30 min at 70 °C. 250 μL of 5 M potassium acetate was added and mixed by pipetting, followed by 15 min incubation on ice. Samples were centrifuged at 14,000 rpm for 20 min and supernatants transferred to a clean tube before isopropanol precipitation (700 μL isopropanol followed by 10 min centrifugation at 14,000 rpm). Pellets were cleaned with 70% ethanol before adding 500 μL TE 10/1 solution (10 mM Tris-HCl pH 7.5, 1 mM EDTA pH 8) followed by 30 min heating at 42 °C and gentle shaking. When DNA was completely dissolved, 50 μL of 3 M sodium acetate were added, followed by a second isopropanol precipitation round (500 μL isopropanol, centrifugation at 14,000 rpm for 15 min). Samples were cleaned in 70% ethanol and pellets dissolved in 50 μL TE 10/1 (10 mM Tris-HCl pH 7.5, 1 mM EDTA pH 8) during incubation at 42 °C for 1 h. DNA concentration was assessed by A280 absorbance on a Nanodrop instrument (Peqlab).

### Engineering primer sequences and cloning

The chromosome coordinates of all conserved RNA folds were retrieved from https://weissmanlab.ucsf.edu/yeaststructures/ and complete sequences extracted using R^50^ and the Bioconductor^51^ packages “BSgenome.Scerevisiae.USCS.sacCer2” and “BSgenome”, which contain the representation of the full genome sacCer2. The resulting multifasta file containing all DNA sequences was parsed and used for forward and reverse amplification primer design taking the first ‘n’ nucleotides from the 5′- or 3′-end until the melting temperature exceeded 58 °C, calculated as 4*(nC + nG) + 2*(nA + nT). Extracted genomic DNA together with the respective primer pairs were used to amplify each RNA fold in a PCR reaction using OneTaq polymerase according to manufacturer’s protocol (New England Biolabs). Successful amplification products were monitored on an agarose gel and were subsequently TA cloned into the pcDNA 3.3 TOPO vector following the manufacturer’s protocol (Invitrogen). Alternatively, for RNA folds failing PCR amplification, a primer extension approach using long primer dimers was employed, followed by TA cloning into pcDNA 3.3 TOPO vector. Correct sequence of all amplicons or chemically synthetized baits was checked by Sanger sequencing. For the GFP reporter screen, a yeast centromeric plasmid backbone was modified to incorporate the fluorescent yeast enhanced GFP (yeGFP) reporter gene driven by the Adh1 promoter and terminator. RNA folds were amplified from the pcDNA 3.3 TOPO plasmid with generic primers that introduced the corresponding restriction enzyme cutting sites for subsequent cloning into the GFP plasmid. According to the RNA fold UTR location, RNA folds were cloned 5′ or 3′ of the GFP reporter with a short linker region; plasmids were transformed into BY4741 yeast strain and selected by the URA3 marker. For the *SFT2* 3′UTR experiments, only the hairpin sequence as previously described^7^ was inserted at the 3′-end of the GFP reporter.

### SILAC labeling of *S. cerevisiae* and extract preparation

Yeast strain YAL6B auxotrophic for arginine and lysine was grown at 30 °C to stationary phase in YPD media and then inoculated 1:10,000 into self-made filter-sterilized SILAC media (6.7 g L^−1^ YNB without amino acids, 2 % Dextrose, 200 mg L^−1^ l-adenine sulfate, 100 mg L^−1^ l-tyrosine, 10 mg L^−1^ l-histidine, 60 mg L^−1^ l-leucine, 10 mg L^−1^ l-methionine, 60 mg L^−1^ l-phenylalanine, 40 mg L^−1^ l-tryptophane, 20 mg L^−1^ Uracil, 20 mg L^−1^ l-arginine (all amino acids from Sigma-Aldrich) and 30 mg L^−1^ of either ‘light’ [^12^C_6_,] or ‘heavy’ [^13^C_6_]l-lysine (Euriso-top). Cells were grown for at least 10 doublings to allow complete labeling and harvested at exponential growth by centrifugation at 20,000 rpm for 45 min at 4 °C (SORVALL ultracentrifuge, Thermo). Two-liter yeast pellets were resuspended in 30 mL lysis buffer (50 mM Tris-HCl pH 7.5, 100 mM NaCl, 1 mM EDTA, 1 mM DTT, 5% glycerol, 1 mM PMSF, 1 μg mL^−1^ Leupeptin, 1 μg mL^−1^ Pepstatin A) and lysed three times at 35,000 psi in a French press at 4 °C. Different batches of lysate preparation were pooled for homogenization and successful incorporation of labeled lysine was checked by mass spectrometry. Extracts for western blotting analysis were prepared in lysis buffer (100 mM NaCl, 50 mM Tris-HCl pH 7.5, 10 mM MgCl_2_, 1 mM PMSF, 0.01% IGEPAL CA-630) by bead milling using 0.5-mm Zirconia/Silica beads in a FastPrep for three cycles of 30 s at 4 °C with 1 min rest in between. Extract protein concentration was determined by Bradford (BioRad).

### RNA transcription and RNA pull-down

The RNA fold baits were created by a PCR reaction with generic amplification primers using the pcDNA 3.3 TOPO plasmid as a template. The forward primer (5′- CGTTAATACGACTCACTATAGGGATCGAACCCTT-3′) incorporated the T7 promoter sequence at the 5′-end of the RNA fold amplicon and the reverse primer (5′- CATGGCCCGGCCCGCGACTATCTTACGCACTTGCATGATTCTGGTCGGTCCCATGGATCCAAAAAAAGATCGAACCCTT-3′) added the S1 minimal aptamer sequence at the 3′-end^9^. PCR products were used for in vitro transcription was performed according to the manufacturer’s protocol (Fermentas) and successful transcription monitored by agarose gel electrophoresis. Tagged RNA oligonucleotides were purified with G-50 micro spin columns (GE Healthcare) and concentration assessed by A280 absorbance on a Nanodrop system (Peqlab). 25 μg of each S1-tagged RNA fold was coupled to paramagnetic streptavidin C1 beads (Dynabeads MyOne, Invitrogen) in RNA-binding buffer (100 mM NaCl, 10 mM MgCl_2_, 50 mM HEPES-KOH pH 7.4, 0.5 % IGEPAL CA-630) and incubated on a rotating wheel for 30 min at 4 °C. RNA-bound beads were washed 3 times with RNA washing buffer (250 mM NaCl, 10 mM MgCl_2_, 50 mM HEPES-KOH pH 7.4, 0.5 % IGEPAL CA-630), followed by incubation with 400 μg yeast extract for 30 min at 4 °C on a rotating wheel. At this point, 650 ng of a competitor mixture containing a pool of all RNA baits without the S1-aptamer tag was added to reduce the number of sticky protein binders. After mild washing, light and heavy fractions were combined and samples were boiled in 1× LDS buffer (Invitrogen) and separated on a 4–12% NuPAGE Novex Bis–Tris precast gel (Life Technologies) at 180 V in 1× MOPS buffer.

### Western blotting analysis

20 μg of whole lysate were run on a 4–12% NuPAGE Novex Bis–Tris precast gel (Life Technologies), transferred to a Protran 85 membrane (Whatman) and probed with either a rabbit TAP antibody (Thermo, 1:1000) or mouse GFP (Roche, 1:1000) as primary antibodies. Either rabbit or mouse HRP-conjugated antibodies (GE Healthcare, 1:2000) were used for detection. Chemiluminiscence detection was done using a SuperSignal West Pico solution (Pierce) and the SeeBlue Plus2 Pre-stained Protein Standard (Thermo Scientific) was used as a marker.

### MS sample preparation and measurement

Coomassie stained gels were cut in one slice and destained with 50% EtOH/25 mM ammonium bicarbonate (ABC). The resulting gel pieces were dehydrated with 100% acetonitrile (ACN) and dried for 5 min in a concentrator (Eppendorf). Samples were incubated with reduction buffer (10 mM DTT/50 mM ABC) for 30 min at 56 °C and further alkylated for 30 min in the dark with iodoacetamide (50 mM IAA/50 mM ABC). Gel pieces were completely dehydrated with ACN and covered in LysC solution (1 μg LysC per sample). Proteins were digested overnight at 37 °C and peptides were extracted twice by incubation with extraction buffer (3% TFA and 30% ACN) for 15 min. The gel pieces were dehydrated with 100% ACN and the extracted volume reduced to ~150 μL in a concentrator (Eppendorf). Extracted peptides were desalted in StageTips^52^ using two layers of C_18_ material (Empore). Eluted peptides were injected via an autosampler into an uHPLC (EASY-nLC 1000, Thermo) and loaded on a 25 cm capillary (75 μm inner diameter; New Objective) packed in-house with Reprosil C18-AQ 1.9 μm resin (Dr. Maisch) for reverse-phase chromatography. The EASY-nLC 1000 HPLC system was directly mounted to a Q Exactive Plus mass spectrometer (Thermo). Peptides were eluted from the column with a 90 min optimized gradient from 2 to 40% ACN with 0.1% formic acid at a flow rate of 200 nL min^−1^. Chromatography was stabilized with a column oven set-up operating at 40 °C (Sonation). The heated capillary temperature was set to 250 °C. Spray voltage ranged from 2.2 to 2.4 kV. The mass spectrometer was operated in data-dependent acquisition mode with one MS full scan and up to ten triggered MS/MS scans using HCD fragmentation^53^. MS full scans were obtained in the orbitrap at 70,000 resolution with a maximal injection time of 20 ms, while MS/MS scan resolution was set to 17,500 resolution and maximal injection for 120 ms. Unassigned and charge state 1 were excluded from MS/MS selection and peptide match was preferred.

### MS data analysis

Raw files were processed with MaxQuant (version 1.5.2.8.)^54^ and searched against *Saccharomyces cerevisiae* Ensembl annotated protein database R64-1-1.24 Oct 2014 (6692 entries) using the Andromeda search engine^55^. Carbamidomethylation was set as a fixed modification, whereas acetyl (N-term protein) and oxidation (Met) were considered as variable modifications. LysC (specific) was selected as enzyme specificity with maximal two miscleavages for MaxQuant analysis. Proteins were quantified with at least 2 ratio counts based on unmodified unique and razor peptides. Known contaminants and reverse hits were removed before plotting the protein ratios of the forward and reverse experiments in R (version 3.2.2). Protein interactors for each RNA fold were identified requiring an enrichment of two-fold in both forward and reverse experiment (log2 SILAC ratios > 1). The enrichment value for each mRNA-protein pair was calculated as the log2 of the Euclidean distance to the origin (coordinates 0,0 in an Euclidean space build upon the dimensions defined by the forward and reverse experiments for each RNA fold). Alternatively, another selection method is proposed to identify interaction partners (Supplementary Fig. 1e): proteins showing enrichment higher than the distance to the origin of a known positive control Puf3.

### GO annotations and RNA-binding domain analysis

Our interactors were cataloged as RNA binding when their associated GO term for Molecular Function (Ensembl version 86, Oct 2016) contained the string RNA binding. For those interactors that did not relate to the term RNA binding, a second classification was done as RNA-related based on previously described RNA-related GO-term annotations^1^. For yet unclassified interactors, their human homologs were classified with the same RNA binding and RNA-related annotation criteria. As a control, proteins from a whole cell lysate measurement were equally classified. For RNA-binding domain classification, a curated list of known PFAM RNA-binding domains^1^ was used (Supplementary Data 3).

### GO annotation of interactors

We performed GO enrichment analysis of binders using SGD Slim Mapper tool. Binders were classified in 3 groups according to the genomic position of the RNA fold they bind to (5′UTR, CDS, 3′UTR).

### Biochemical properties analysis of our RBP set

A peptide properties table containing information about Molecular Weight, Isoelectric Point, Protein Length, Hydropathicity GRAVY Scores, Aromaticity Score (frequency of aromatic amino acids: Phe, Tyr, Trp), Codon Adaptation Index, Codon Bias, FOP Score (Frequency of Optimal Codons), Instability Index and Aliphatic Index, was downloaded from the Saccharomyces Genome Database^56^. Significant differences of our RBP set against two control groups containing all known proteins of the yeast genome (predicted proteome) and our measured proteome were calculated with a Student’s *t*-test (*p*-value corrected for multiple testing, FDR).

### Disordered region analysis

Complete peptide sequences for our interactor set were retrieved from Ensembl version 86 (Oct 2016) and used for disordered region probability calculation with IUPred2A^57^, defined as a lack of known tertiary structure under native conditions. The default prediction type for long disorder region was used and a score based on the percentage of bases with disorder probability higher than 50% was calculated for each protein interactor.

### Genetic interaction data integration and network analysis

For genetic interaction data integration, a recent large-scale genetic interaction study^34^ was used. For genetic interaction scores (|E|) higher than 0.08 between our RNA folds and protein interactors, an annotation matrix was calculated depicting the reported |E|scores. Pearson correlation coefficients (PCC) of genetic interaction profiles for all relevant genes (including protein interactors and their mRNA targets) were calculated. Genes with similar genetic interaction landscape were identified using a simple network analysis: each gene was a vertex, and an edge between 2 vertices was defined if the PCC between these two proteins was higher than 0.2, as described in Costanzo et al. Community structure on the network was inferred using a method implemented in the igraph package^58^ based on propagating labels, which works by assigning vertices to unique communities and then updates those communities by doing majority voting around a vertex.

### PAR-CLIP data validation

PAR-CLIP raw data for Nab2 (GSM1442550), Pab1 (GSM1442553) and Yra1 (GSM1442559) were downloaded from GSE59676^59^. Reads were preprocessed with the Fastx toolkit (http://hannonlab.cshl.edu/fastx_toolkit/) to remove adapter sequences, keep sequences longer than 15 nucleotides, filter artifacts and remove low quality reads (Phread scores < 23). Preprocessed reads were then aligned onto the sacCer3 reference genome with Bowtie^60^ version 1.1.2 and options -q -p 8 -S -v 2 -m 10–best–strata to allow up to 2 mismatches and reads mapping to up to 10 loci, keeping only alignments in the best stratum. Scaled BigWig tracks were generated from the alignment files. To call peaks, the Piranha^61^ 1.2.1 peak caller was used with options -b 50 -d Poisson, which bins reads into bins of 50 bp and uses the Poisson distribution to model the counts. Peaks called with Benjamin and Hochberg (BH) corrected *p*-value below 0.05 were considered true peaks.

### Yeast tagged- and knock-out strains

Yeast knock-out and TAP-tagged strains used for pSILAC, WB and RIP experiments are listed in Supplementary Data 5. All strains were validated by PCR prior to experiments.

### RNA immunoprecipitation (RIP) analysis

TAP-tagged strains from Dharmacon collection were used for TAP-RIP experiments. 100–150 mL of exponentially growing cultures were crosslinked for 10 min with 1.2% formaldehyde (Applichem) after cell number normalization. Samples were quenched with glycine (360 mM, Applichem) for 5 min at room temperature. After cooling down to 4 °C on ice for 15 min, cells were pelleted at 4 °C by centrifugation (1731 rcf, 3 min), washed twice with ice-cold PBS and stored at −80 °C until processing. Cell pellets were lysed in FA buffer (50 mM HEPES-KOH pH 7.5, 140 mM NaCl, 1 mM EDTA pH 8, 1% Triton X-100, protease inhibitor cocktail (Roche) via two 30 s rounds of 6.5 M s^−1^ FastPrep (MP Biomedical). Samples were diluted in FA buffer supplemented with 0.1% sodium-deoxycholate (SOD). Soluble and chromatin extracts were separated by centrifugation (7 min at 17949 rcf). Subsequently, 2 mg of soluble extracts were incubated overnight at 4 °C with 75 µL of pre-washed IgG Beads (GE Healthcare) with 5% BSA. 50 µL of extracts was separated as an input control. Beads were washed with 1 mL of FA buffer, Buffer 500 (50 mM HEPES-KOH pH 7.5, 500 mM NaCl, 1 mM EDTA pH 8, 1% Triton X-100, 0.1% SOD), Buffer III (10 mM Tris-HCl pH 8, 1 mM EDTA pH 8, 150 mM LiCl, 1% NP40, 1% SOD) and TE buffer (100 mM Tris-HCl pH 8, 50 mM EDTA pH 8) at 4 °C with 5 min incubation times between washes. Proteins were eluted with Elution Buffer (50 mM Tris-HCl pH 7.5, 1% SDS, 10 mM EDTA pH 8) twice for 8 min at 65 °C. Samples were de-crosslinked for 2 h at 65 °C and subsequently digested with 3 units of DNase I (QIAGEN) for 2 h at 37 °C. After digestion, eluted samples were digested with proteinase K (0.75 mg mL^−1^) for 2 h at 65 °C. RNA samples were purified using the RNeasy MinElute Cleanup kit (QIAGEN). Purified RNA samples were digested once more with 3 units of DNase I (QIAGEN) and purified. RNA samples were subjected to reverse transcription before quantification by qPCR for different loci. RNA samples were split into 2 fractions. One fraction was used to measure the RNA levels and the other fraction was used as a negative control of reverse transcription (no reverse transcriptase added). The RNA was incubated at 90 °C for 1 min with 0.4 µL 25 mM dNTPs, 0.8 µL 5 µM of different primer pairs in 10 µL final volume. The RNA was then cooled down to 55 °C at a 0.8 °C s^−1^ temperature rate. A mix of 1 µL 100 mM DTT, 1 µL SuperScript III in 1× FS-buffer (Invitrogen) was added to the reactions. Negative control sample did not contain SuperScript III reverse transcriptase. The RNA was reverse transcribed for 60 min at 55 °C. The enzyme was inactivated at 70 °C for 15 min. RNA samples were diluted with 30 µL H_2_O and used in qPCR for quantification.

### RNA quantification by RT-qPCR

Exponentially growing cells were collected and resuspended in 400 µL AE Buffer (50 mM sodium citrate in 10 mM EDTA pH5.3) and lysed with 500 µL calibrated phenol (with AE buffer) at 65 °C for 5 min. The aqueous phase was separated by centrifugation and mixed with 500 µL phenol–chloroform–isoamyl alcohol for 5 min at room temperature. The aqueous phase was again separated by centrifugation and collected. RNA was precipitated with 40 µL 3 M sodium acetate and 1 mL 100% ethanol. RNA was pelleted by centrifugation and washed with 80% ethanol. Air-dried pellet was subsequently resuspended in a solution containing 3 µL DNase I (QIAGEN) in RDD buffer to digest genomic DNA. DNA was digested for 45 min at 37 °C and remaining RNA was purified with RNeasy MinElute Cleanup kit (QIAGEN). The RNA was incubated at 90 °C for 1 min with 0.4 µL 25 mM dNTPs, 0.8 µL 5 µM of different primer pairs in 10 µL final volume reaction. The RNA was then cooled down to 55 °C at a 0.8 °C s^−1^ temperature rate. A mix of 1 µL 100 mM DTT, 1 µL SuperScript III in 1× FS-buffer (Invitrogen) was added to the reactions. Negative control sample did not contain SuperScript III reverse transcriptase. The RNA was reverse transcribed for 60 min at 55 °C. The enzyme was inactivated at 70 °C for 15 min. RNA samples were diluted with 30 µL H_2_O and subjected to qPCR. Data were processed according to the 2^(−ΔΔCt)^ method and expressed as %input. For both ±RT conditions, %input was calculated normalized to the +RT input (5%) values. Finally, +RT %input were normalized to the respective −RT %input. Error bars show SEM, calculated as SEM = (SEM_1_^2^ + SEM_2_^2^)^1/2^.

### Yeast transformation

Exponentially growing BY4742 yeast cells in YPD medium were pelleted (300×*g*, 5 min) and gently resuspended in 3 mL SORB buffer (100 mM LiOAc, 10 mM Tris-HCl pH 8.0, 1 mM EDTA pH 8.0, 1 M Sorbitol). 50 μg of sheared salmon sperm (Ambion) carrier DNA was boiled 5 min at 95 °C prior to the addition of 100 ng of plasmid DNA. Cells were resuspended in 100 μL LiT solution (100 mM LiAc, 10 mM Tris-HCl pH 7.4), plasmid-carrier DNA was added and followed by the addition of 500 μL of PEG/LiT (polyethylene glycol 3350 (Sigma-Aldrich)). Samples were vortexed and incubated in a rotation wheel for 30 min at room temperature. 50 μL of DMSO (Sigma-Aldrich) were added and incubated for 15 min at 42 °C. Cells were pelleted (500×*g* for 30 s) and resuspended in 200 μL of SD-URA medium, incubated at 30 °C for 30 min and plated on SD-URA plates for 2–3 days.

### Flow cytometry

The relevant RNA folds were cloned into the corresponding UTR location of a centromeric GFP reporter plasmid. The resulting plasmids were used in the respective yeast knock-out strains (Dharmacon). The BY4741 yeast knock-out strains transformed with the GFP reporter plasmid were grown to saturation in SD-URA selection media at 30 °C, diluted 1:100 and further grown to OD_600nm_ of 0.7–0.9. Cells were analyzed by flow cytometry on a BD LSRFortessa SORP (BD Biosciences). Doublets were excluded via SSC-W signal and dead cells were excluded by DAPI staining. 20,000 events were measured per experiment and median values used for data analysis with FlowJo software (v10.5.3). GFP mean fluorescence intensities for the knock-out experiments were normalized to the corresponding wild-type condition and the mean value of three experimental replicates was plotted. Error bars represent the standard deviation of the GFP knock-out/wild-type values.

### Pulsed SILAC

SILAC medium (6.7 g YNB w/o amino acids and with ammonium sulfate, 20 g Dextrose, 0.2 g l-adenine sulfate, 0.1 g l-tyrosine, 0.01 g l-histidine, 0.06 g l-leucine, 0.01 g l-methionine, 0.06 g l-phenylalanine, 0.04 g l-tryptophane, 0.02 g uracil and 0.02 g l-arginine per liter) was supplemented with 30 mg L^−1^ either lysine-0, lysine-4 and lysine-8 (Eurisotop) and sterile filtered through a 0.22-μm filter (Fisher Science). A preculture of 5 mL in lysine-0 medium was grown overnight at 30 °C. The culture was diluted and grown to OD_600_ = 0.4 in lysine-0 medium, prior to two washes with PBS (cells pelleted by centrifugation at 500 g). When cells were resuspended in 15 mL lysine-8 medium (knock-out strain) and lysine-4 medium (wild-type strain), actinomycin D was added at a final concentration of 1 μg mL^−1^. Cells were grown with gentle agitation for 2 h at 30 °C. Cells from both cultures were mixed at OD_600_ = 0.5 and harvested by centrifugation at 14,000×*g* for 2 min at 4 °C. The pellet was washed with PBS and transferred to a clean tube. The cells were again centrifuged at 14,000×*g* for 15 s at 4 °C and resupended in 50 μL 1× NuPAGE LDS buffer (Thermo), sonicated for 10 cycles (30 s ON/OFF), spun down at 14.000×*g* and 20 μL loaded in a 10% NuPage NOVEX precast gel (Thermo). Subsequent protein separation, staining and in-gel digest was done as described (see MS sample preparation and measurement section). Data were analyzed with MaxQuant and further processed with in-house scripts (see MS data analysis).

### Reporting summary

Further information on research design is available in the Nature Research Reporting Summary linked to this article.

## Supplementary information

Supplementary Information

Reporting Summary

Description of Additional Supplementary Files

Supplementary Data 1

Supplementary Data 2

Supplementary Data 3

Supplementary Data 4

Supplementary Data 5

Supplementary Data 6

Supplementary Data 7

## Data Availability

The data that support this study are available from the corresponding author upon reasonable request. The source data underlying Figs. 3b, c and 4b, e are provided as a Source Data file. The mass spectrometry raw data are available at ProteomeXchange (http://www.proteomeexchange.org) under the dataset identifier PXD014092.

## Source data

Source Data

## Code availability

Custom code used to analyze data in this study is available at GitHub https://github.com/ssayols/rnafold_interactome_casas_vila_et_al.
