# Supplementary material for: The RNA fold interactome of evolutionary conserved RNA structures in S. cerevisiae
Source: Nat Commun. 2020 Jun 3;11:2789. doi: 10.1038/s41467-020-16555-4 (PMC7270185; doi:10.1038/s41467-020-16555-4)
Supplement: Supplementary file 1 — Supplementary Information [file 41467_2020_16555_MOESM1_ESM.pdf]

**Supplementary Information**

**The RNA fold interactome of evolutionary conserved RNA structures in *S. cerevisiae***

Casas-Vila et al.

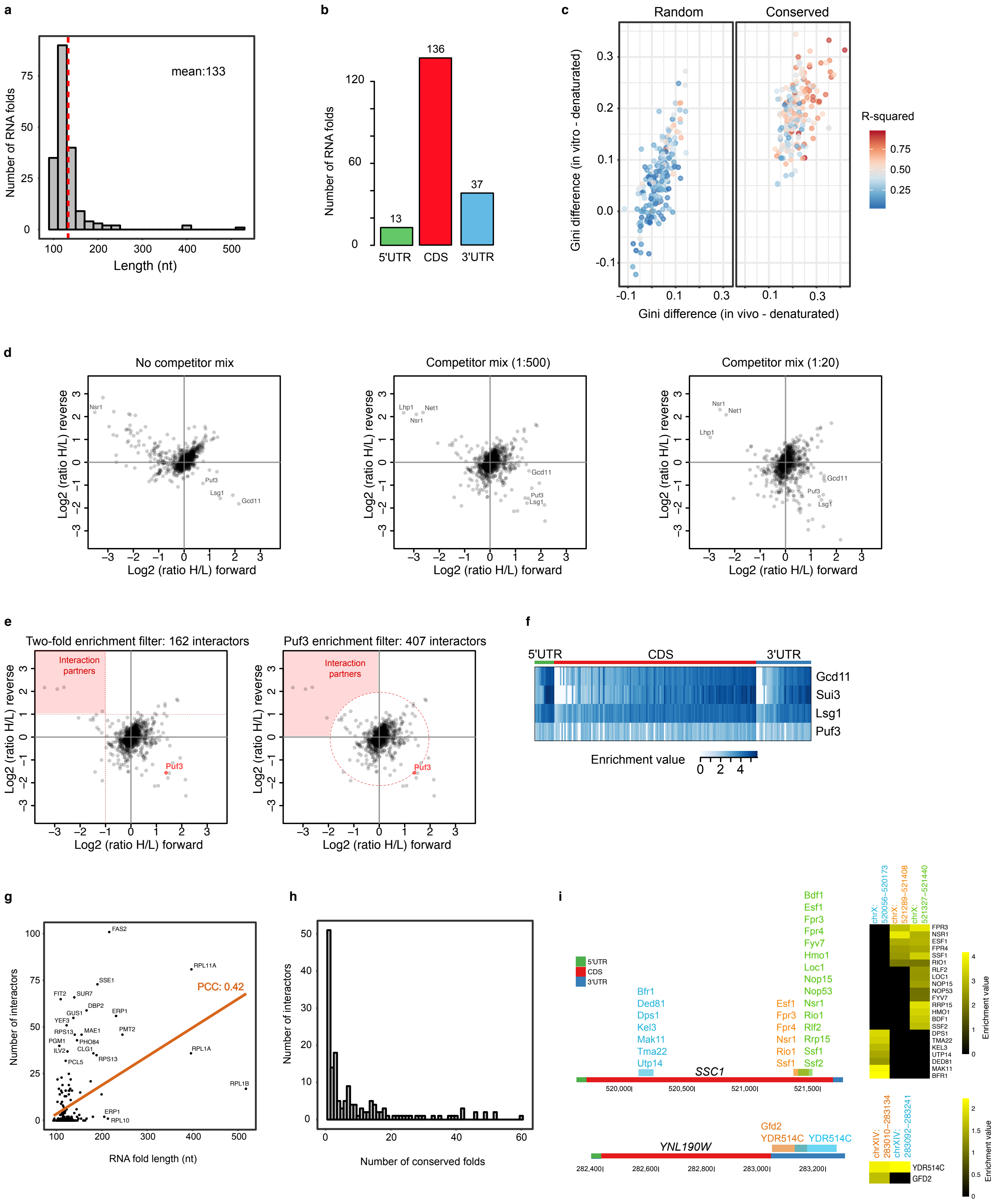


**Supplementary Figure 1. RNA fold features and technical aspects of our RNA fold interactome.**

**(a)** Length (nt) distribution of the 188 evolutionary conserved RNA folds. Red-dashed line indicates mean value.

**(b)** Distribution of the conserved RNA folds according to location (5'UTR, CDS, 3'UTR).

**(c)** Structure analysis based on *in vitro* and *in vivo* DMS-Seq data comparing 188 random genomic regions to the set of 188 evolutionary conserved RNA structures. Folds are colored according to *in vitro*/*in vivo* DMS-Seq correlations. R-squared coloring according to the degree of correlation between *in vivo* and *in vitro* conditions.

**(d)** Two-dimensional interaction plot showing protein enrichment for one RNA fold using different competitor mix concentrations (0, 1:500 and 1:20 dilutions).

**(e)** Examples of two different filtering criteria to score for interaction partners. Red area indicates interaction partners.

**(f)** Enrichment values for the four control proteins Puf3, Gcd11, Sui3 and Lsg1 at the *COX17* UTR in all 186 pull-down experiments.

**(g)** Correlation plot on number of RBP candidates binding one structure vs. structure length. PCC: Pearson Correlation Coefficient.

**(h)** Histogram showing the number of RBP interactors binding to each RNA fold.

**(i)** Schematics of RNA fold localization on the *SSC1* and *YNL190W* mRNAs harboring multiple folds and the corresponding interactors. Enrichment values for each interaction are shown in the heatmap.

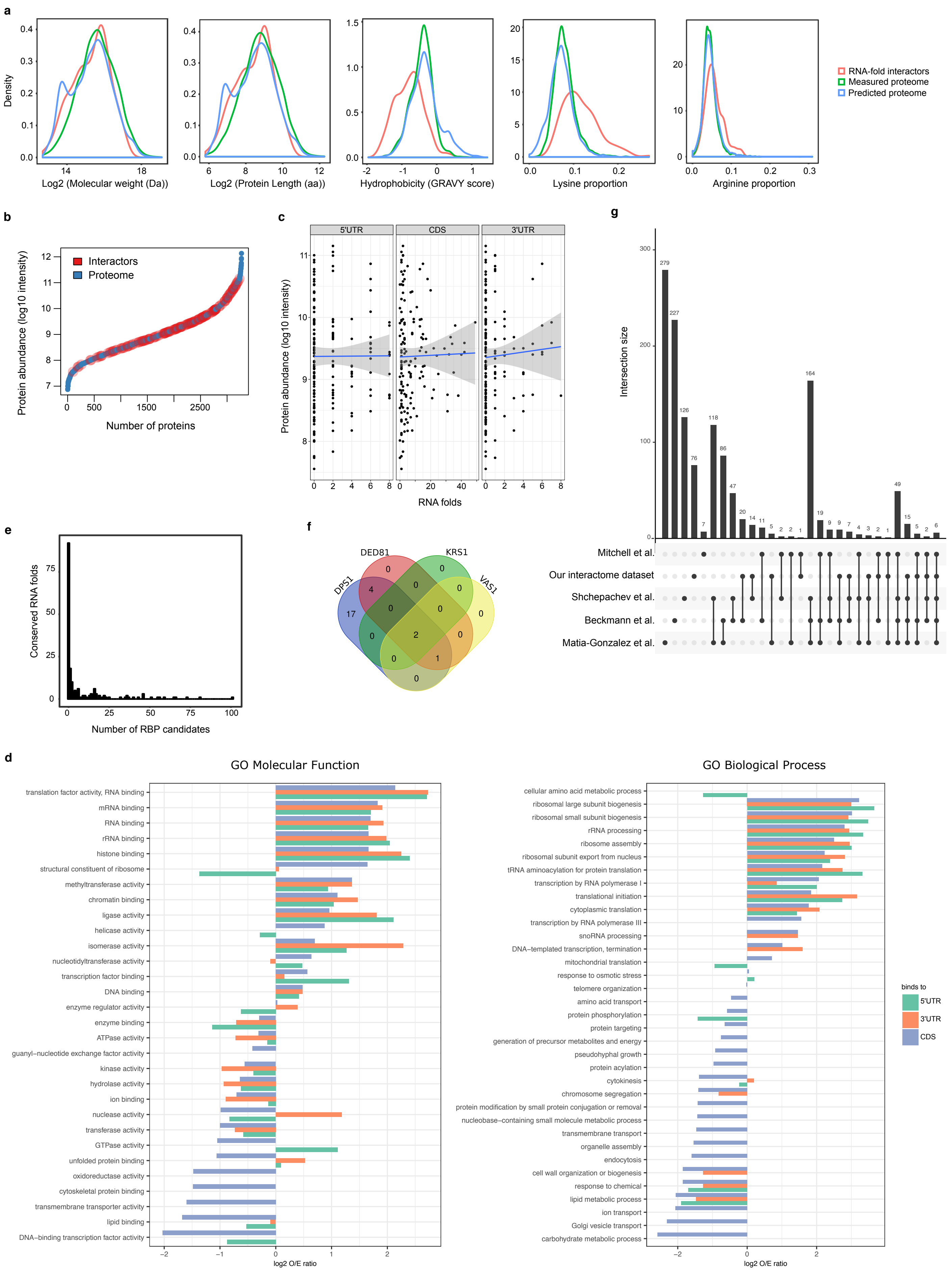
a
Density
Log2 (Molecular weight (Da))
Log2 (Protein Length (aa))
Hydrophobicity (GRAVY score)
Lysine proportion
Arginine proportion
RNA-fold interactors
Measured proteome
Predicted proteome
b
Protein abundance (log10 intensity)
Interactors
Proteome
Number of proteins
c
5'UTR
CDS
3'UTR
Protein abundance (log10 intensity)
RNA folds
g
Intersection size
Mitchell et al.
Our interactome dataset
Shchepachev et al.
Beckmann et al.
Matia-Gonzalez et al.
e
Conserved RNA folds
Number of RBP candidates
f
DED81
KRS1
DPS1
VAS1
d
GO Molecular Function
translation factor activity, RNA binding
mRNA binding
RNA binding
rRNA binding
histone binding
structural constituent of ribosome
methyltransferase activity
chromatin binding
ligase activity
helicase activity
isomerase activity
nucleotidyltransferase activity
transcription factor binding
DNA binding
enzyme regulator activity
enzyme binding
ATPase activity
guanyl−nucleotide exchange factor activity
kinase activity
hydrolase activity
ion binding
nuclease activity
transferase activity
GTPase activity
unfolded protein binding
oxidoreductase activity
cytoskeletal protein binding
transmembrane transporter activity
lipid binding
DNA−binding transcription factor activity
log2 O/E ratio
GO Biological Process
cellular amino acid metabolic process
ribosomal large subunit biogenesis
ribosomal small subunit biogenesis
rRNA processing
ribosome assembly
ribosomal subunit export from nucleus
tRNA aminoacylation for protein translation
transcription by RNA polymerase I
translational initiation
cytoplasmic translation
transcription by RNA polymerase III
snoRNA processing
DNA−templated transcription, termination
mitochondrial translation
response to osmotic stress
telomere organization
amino acid transport
protein phosphorylation
protein targeting
generation of precursor metabolites and energy
pseudohyphal growth
protein acylation
cytokinesis
chromosome segregation
protein modification by small protein conjugation or removal
nucleobase−containing small molecule metabolic process
transmembrane transport
organelle assembly
endocytosis
cell wall organization or biogenesis
response to chemical
lipid metabolic process
ion transport
Golgi vesicle transport
carbohydrate metabolic process
log2 O/E ratio
binds to
5'UTR
3'UTR
CDS

**Supplementary Figure 2. Characterization of our RNA fold interactors and comparison to other studies.**

**(a)** Density plots showing biochemical features of our RNA fold interactome (orange), measured proteome (green) and predicted proteome (blue).

**(b)** Ranked protein abundance plotted against enriched interactors reveals that interactors (red) cover the full proteome abundance range.

**(c)** Protein abundance grouped by fold type (5'UTR, CDS, 3'UTR). Shaded area represents confidence interval (0.95) around the linear model.

**(d)** GO term enrichment analysis on molecular function and biological process of interactors differentiated by location within the target RNA (5'UTR, CDS, 3'UTR). The log2 values of the observed/expected ratio for the top25 GO terms of ranked by fold type are shown.

**(e)** Histogram showing the number of conserved folds bound by each RBP candidate.

**(f)** Overlap of target genes for the four identified tRNA synthetases.

**(g)** Comparison of our RBP candidates with other existing interactome capture studies in *S. cerevisiae*.

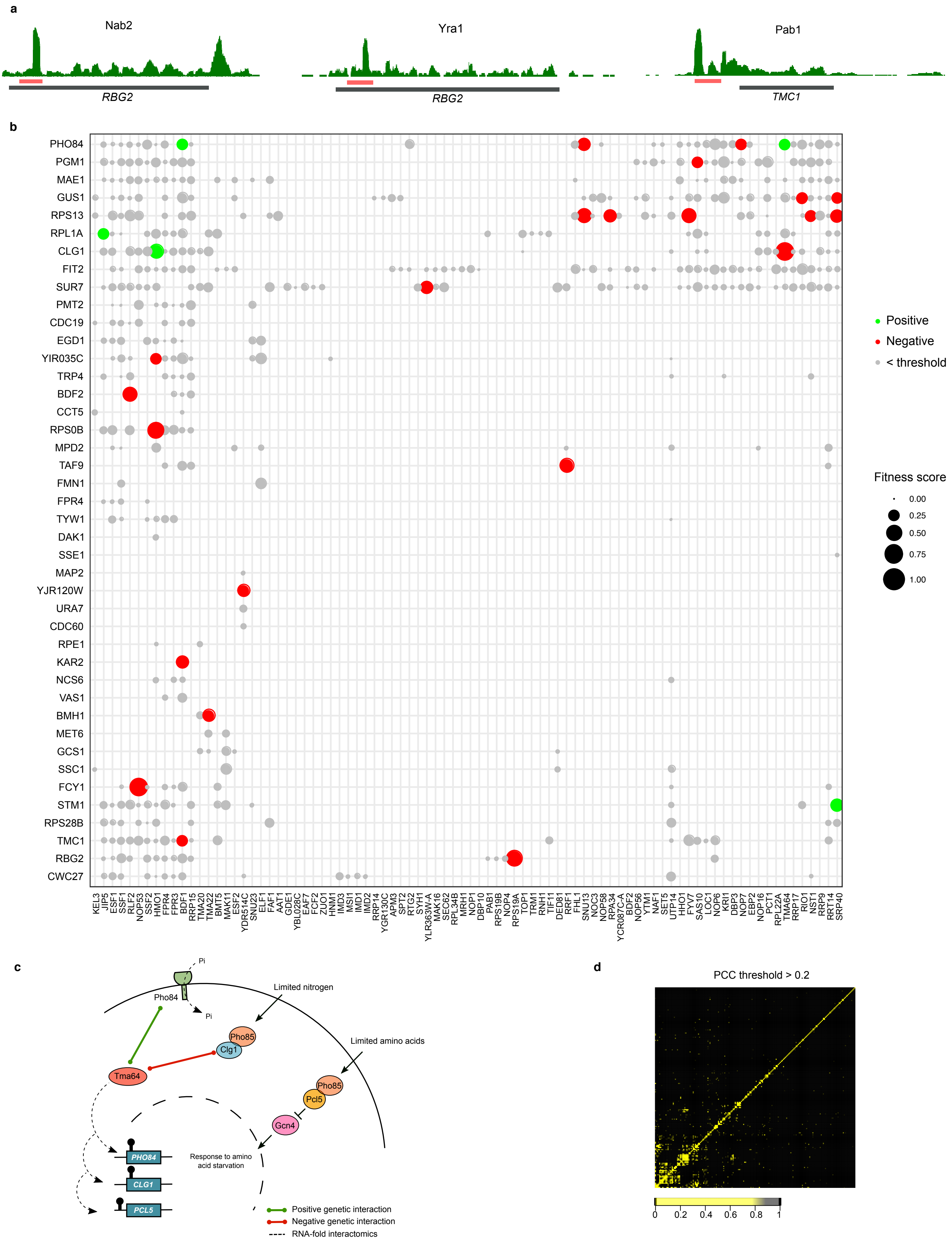

a
Nab2
RBG2
Yra1
RBG2
Pab1
TMC1
b
PHO84
PGM1
MAE1
GUS1
RPS13
RPL1A
CLG1
FIT2
SUR7
PMT2
CDC19
EGD1
YIR035C
TRP4
BDF2
CCT5
RPS0B
MPD2
TAF9
FMN1
FPR4
TYW1
DAK1
SSE1
MAP2
YJR120W
URA7
CDC60
RPE1
KAR2
NCS6
VAS1
BMH1
MET6
GCS1
SSC1
FCY1
STM1
RPS28B
TMC1
RBG2
CWC27
KEL3
JIP5
ESF1
SSF1
RLF2
NOP53
SSF2
HMO1
FPR4
FPR3
BDF1
RRP15
TMA20
TMA22
BMT5
MAK11
ESF2
YDR514C
SNU23
ELF1
FAF1
AAT1
GDE1
YBL028C
EAF7
FCF2
ZUO1
HNM1
IMD3
MSI1
IMD1
IMD2
RRP14
YGR130C
APM3
SPT2
RTG2
SYH1
YLR363W-A
MAK16
SEC62
RPL34B
MRH1
NOP1
DBP10
PAB1
RPS19B
NOP4
RPS19A
TOP1
TRM1
RNH1
TIF11
DED81
RRF1
FHL1
SNU13
NOC3
NOP58
RPA34
YCR087C-A
BDF2
NOP56
YTM1
NAF1
SET5
UTP14
HHO1
FYV7
SAS10
LOC1
NOP6
KRI1
DBP3
NOP7
EBP2
NOP16
PCT1
RPL22A
TMA64
RRP17
RIO1
NST1
RRP9
RRT14
SRP40
Positive
Negative
< threshold
Fitness score
0.00
0.25
0.50
0.75
1.00
c
Pi
Pho84
Pi
Limited nitrogen
Pho85
Clg1
Limited amino acids
Tma64
Pho85
Pcl5
Gcn4
Response to amino acid starvation
PHO84
CLG1
PCL5
Positive genetic interaction
Negative genetic interaction
RNA-fold interactomics
d
PCC threshold > 0.2
0
0.2
0.4
0.6
0.8
1

**Supplementary Figure 3. RNA fold-protein interaction validation and genetic interaction data integration.**

**(a)** PAR-CLIP data for Pab1, Yra1 and Nab2 validates binding to the investigated RNA folds. The location of the corresponding evolutionary conserved RNA fold is indicated in salmon.

**(b)** Complete matrix of genetic interactions described for our RBP (x-axis) and RNA fold (y-axis) interacting pairs. Genetic interactions with a fitness score > 0.08 are colored according to positive (green) and negative (red) interactions and the remaining are shown in grey. The circle size is proportional to the fitness score of the double knock-out strain of the two relevant genes.

**(c)** Integration of literature knowledge of Tma64 and functional evidences based on our interactomics screen and genetic interaction data.

**(d)** Distance correlation of genetic interaction profiles of all genes (interactors and mRNAs). Correlations higher than 0.2 are highlighted in yellow and filtered for similar genetic interaction profiles.

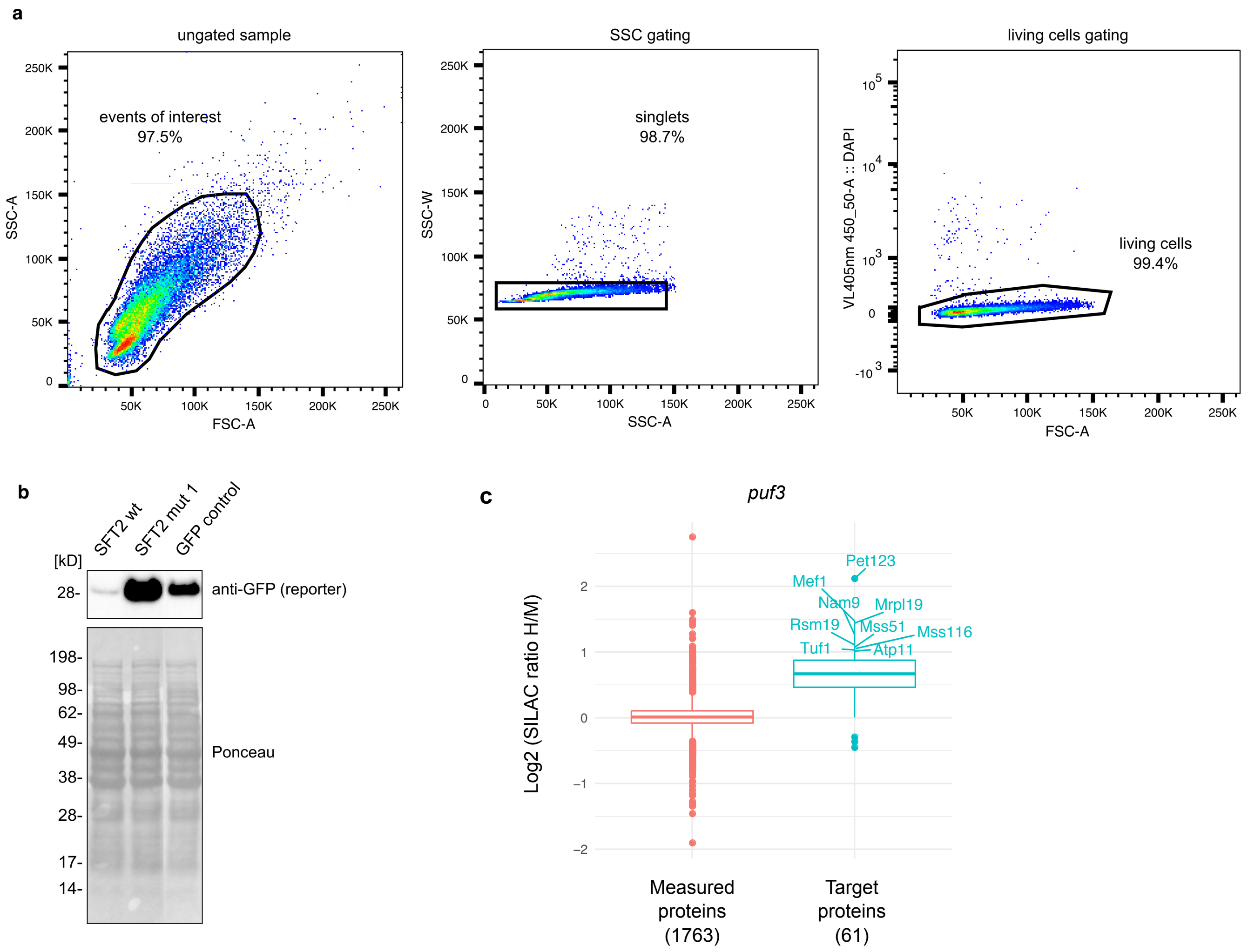


**Supplementary Figure 4. Technical aspects of the reporter screen and positive control for pulsed SILAC.**

**(a)** Example of gating used for GFP fluorescence intensities quantification by flow cytometry.

**(b)** Immunostaining shows changes in GFP levels upon fusion of the wild-type or the mutant 5'UTR *SFT2* loop as previously validated[7], and examined in two independent biological experiments.

**(c)** Pulsed SILAC box plots for *puf3* knock-out compared to a wild-type strain. Known target genes (blue) are upregulated compared to all measured proteins (salmon)[40]. Boxes show median (center) and interquartile ranges (ends), lower whisker representing the smallest observation greater than or equal to 1.5 times the interquartile range and upper whisker representing the largest observation less than or equal to 1.5 times the interquartile range.
