# Supplementary material for: The RNA fold interactome of evolutionary conserved RNA structures in S. cerevisiae
Source: Nat Commun. 2020 Jun 3;11:2789. doi: 10.1038/s41467-020-16555-4 (PMC7270185; doi:10.1038/s41467-020-16555-4)
Supplement: Supplementary file 2 — Reporting Summary [file 41467_2020_16555_MOESM2_ESM.pdf]

nature research

Corresponding author(s): Falk Butter

Last updated by author(s): Apr 8, 2020

Reporting Summary

Nature Research wishes to improve the reproducibility of the work that we publish. This form provides structure for consistency and transparency in reporting. For further information on Nature Research policies, see Authors & Referees and the Editorial Policy Checklist.

Statistics

For all statistical analyses, confirm that the following items are present in the figure legend, table legend, main text, or Methods section.

n/a	Confirmed	
☐	☒	The exact sample size (n) for each experimental group/condition, given as a discrete number and unit of measurement
☐	☒	A statement on whether measurements were taken from distinct samples or whether the same sample was measured repeatedly
☐	☒	The statistical test(s) used AND whether they are one- or two-sided<br>*Only common tests should be described solely by name; describe more complex techniques in the Methods section.*
☐	☒	A description of all covariates tested
☐	☒	A description of any assumptions or corrections, such as tests of normality and adjustment for multiple comparisons
☐	☒	A full description of the statistical parameters including central tendency (e.g. means) or other basic estimates (e.g. regression coefficient) AND variation (e.g. standard deviation) or associated estimates of uncertainty (e.g. confidence intervals)
☐	☒	For null hypothesis testing, the test statistic (e.g. F, t, r) with confidence intervals, effect sizes, degrees of freedom and P value noted<br>*Give P values as exact values whenever suitable.*
☒	☐	For Bayesian analysis, information on the choice of priors and Markov chain Monte Carlo settings
☐	☒	For hierarchical and complex designs, identification of the appropriate level for tests and full reporting of outcomes
☒	☐	Estimates of effect sizes (e.g. Cohen's d, Pearson's r), indicating how they were calculated

Our web collection on statistics for biologists contains articles on many of the points above.

Software and code

Policy information about availability of computer code

Data collection	MaxQuant quantitative proteomics software version 1.5.2.8 (Olsen, J. V, et al. 2007).<br>FlowJo software version 10.5.3 for cytometry data analysis. ClustalW version 1.81. RNAalifold from Vienna RNA package version 2.2.8.<br>IUPred2A version 17 December 2018. Bowtie version 1.1.2. Piranha version 1.2.1.
Data analysis	All custom code used to analyze data in this study is available at https://github.com/ssayols/rnafold_interactome_casas_vila_et_al and described in details in the Methods section.

For manuscripts utilizing custom algorithms or software that are central to the research but not yet described in published literature, software must be made available to editors/reviewers. We strongly encourage code deposition in a community repository (e.g. GitHub). See the Nature Research guidelines for submitting code & software for further information.

Data

Policy information about availability of data

All manuscripts must include a data availability statement. This statement should provide the following information, where applicable:

- Accession codes, unique identifiers, or web links for publicly available datasets
- A list of figures that have associated raw data
- A description of any restrictions on data availability

Mass spectrometry raw data files have been deposited in ProteomeXchange under the accession identifier: PXD014092. The source data underlying Figures 3b, 3c, 4b and 4e are provided as a Source Data file.

Field-specific reporting

Please select the one below that is the best fit for your research. If you are not sure, read the appropriate sections before making your selection.

☒ Life sciences ☐ Behavioural & social sciences ☐ Ecological, evolutionary & environmental sciences

For a reference copy of the document with all sections, see nature.com/documents/nr-reporting-summary-flat.pdf

Life sciences study design

All studies must disclose on these points even when the disclosure is negative.

Sample size	For this type of high-throughput experiments, there are important constrains in terms of time and cost. Therefore, in the interactomics SILAC screen, two replicates were performed (forward and reverse) which is a well-accepted approach in the proteomics field. Functional hit validation data in yeast cells was derived from at least three samples per condition, which is a well-accepted setup for reporter protein-based experiments.
Data exclusions	No data were excluded from analysis
Replication	For the RNA fold interactomics screen, experiments were performed in duplicates (forward and reverse experimental approach). Cytometry experiments were performed twice independently, each time in three experimental replicates. Results were consistent and only one batch is plotted in triplicates. All RIP experiments included in the paper were performed in three technical replicates and pulsed SILAC experiments in three biological replicates. Additional measures were taken to verify reproducibility in the interactomics SILAC screen and experiments showing large deviations between the forward and reverse pull-downs were repeated (both forward and reverse experiments).
Randomization	Transcribed RNA folds were allocated randomly in 96-well plates and the same order was used for MS measurement of our interactomics SILAC screen.
Blinding	RNA folds were coded according to unique identifiers based on plate and well positions and decoded to gene names once all data were collected. However, blinding was not relevant to the study because the selection of protein hits was purely quantitative based on our quantitative proteomics data.

Reporting for specific materials, systems and methods

We require information from authors about some types of materials, experimental systems and methods used in many studies. Here, indicate whether each material, system or method listed is relevant to your study. If you are not sure if a list item applies to your research, read the appropriate section before selecting a response.

Materials & experimental systems

n/a	Involved in the study	
☐	☒	Antibodies
☒	☐	Eukaryotic cell lines
☒	☐	Palaeontology
☒	☐	Animals and other organisms
☒	☐	Human research participants
☒	☐	Clinical data

Methods

n/a	Involved in the study	
☒	☐	ChIP-seq
☐	☒	Flow cytometry
☒	☐	MRI-based neuroimaging

Antibodies

Antibodies used	Polyclonal Rabbit TAP-tagged Antibody from Invitrogen (Thermo Fisher Scientific distributed), catalogue number: CAB1001. Dilution used 1:1000.<br>Monoclonal Mouse anti-GFP antibody from Roche (Sigma-Aldrich distributed), catalogue number: 11814460001, clones 7.1 and 13.1, dilution 1:1000.<br>Donkey anti-rabbit IgG linked to horse-radish peroxidase from GE Healthcare (catalogue number: NA934V) and sheep anti-mouse IgG coupled to horse-radish peroxidase from GE Healthcare (catalogue number: NA931V) were used as secondary antibodies, both at 1:2000 dilution.
Validation	Monoclonal Mouse anti-GFP antibody quality control by Sigma-Aldrich: E.coli extract containing a recombinant GFP fusion protein showed a specific band at a 0.4 ug/ml antibody concentration, whereas no band was visualized for a negative control without GFP fusion protein.<br>TAP antibody testing data is available for two TAP-tagged proteins at the manufacturer's website (https://www.thermofisher.com/antibody/product/TAP-Tag-Antibody-Polyclonal/CAB1001).

Flow Cytometry

Plots

Confirm that:

☒ The axis labels state the marker and fluorochrome used (e.g. CD4-FITC).

☒ The axis scales are clearly visible. Include numbers along axes only for bottom left plot of group (a 'group' is an analysis of identical markers).

☒ All plots are contour plots with outliers or pseudocolor plots.

☒ A numerical value for number of cells or percentage (with statistics) is provided.

Methodology

Sample preparation	BY4741 yeast cells were grown in SD-URA media to a D600nm of 0.7 - 0.9. Cells were diluted 1:20 in fresh SD-URA media into a 96-well plate and DAPI stained (final concentration 0.5 ug/ml). Measurements were performed using the high-throughput sampler.
Instrument	LSRFortessa SORP (Special Order Research Product) from BD Biosciences
Software	FlowJo version 10.5.3 was used for analysis
Cell population abundance	> 95% of all events were used for FSC/SSC gating. After gating for living cells, approximately the 80% of the initial population was used for GFP fluorescence intensity analysis
Gating strategy	Gating for single cell events were established based on SSC pulse width (SSC-W). Next, living cells were gated based on DAPI exclusion (DAPI: Excitation 405 nm laser, detection 450/50 BP filter). GFP (Excitation 488 nm laser, detection 530/30 BP filter) positive cell populations were established as compared to an untransformed negative control as shown in supplemental figure 4a. GFP median fluorescence intensities of the living cell population were used for quantification.

☒ Tick this box to confirm that a figure exemplifying the gating strategy is provided in the Supplementary Information.
