# Supplementary material for: The RNA fold interactome of evolutionary conserved RNA structures in S. cerevisiae
Source: Nat Commun. 2020 Jun 3;11:2789. doi: 10.1038/s41467-020-16555-4 (PMC7270185; doi:10.1038/s41467-020-16555-4)
Supplement: Supplementary file 3 — Description of Additional Supplementary Files [file 41467_2020_16555_MOESM3_ESM.pdf]

Description of Additional Supplementary Files

File Name: Supplementary Data 1
Description: List of protein interactors identified by MaxQuant analysis.

File Name: Supplementary Data 2
Description: List of mRNA-protein interactors in the heatmap of Figure 1d, showing the enrichment value for each scored mRNA-protein pair.

File Name: Supplementary Data 3
Description: List of GO terms and RNA-binding domains used in our interactor set analysis.

File Name: Supplementary Data 4
Description: GO term enrichment analysis of similar genetic interaction profile communities of our protein interactors and their mRNA target genes.

File Name: Supplementary Data 5
Description: List of primers and yeast strains used in this study.

File Name: Supplementary Data 6
Description: 162 interactors ranked by fold type and GO term enrichment analysis on 5'UTR, CDS and 3'UTR interactors.

File Name: Supplementary Data 7
Description: Pulsed SILAC results for puf3, nsr1 and tma20.
